# Activation of the NLRP3 Inflammasome by Particles from the Echinococcus granulosus Laminated Layer

**DOI:** 10.1128/IAI.00190-20

**Published:** 2020-08-19

**Authors:** Cecilia Casaravilla, Álvaro Pittini, Dominik Rückerl, Judith E. Allen, Álvaro Díaz

**Affiliations:** a Área Inmunología, Departamento de Biociencias (Facultad de Química) and Cátedra de Inmunología, Instituto de Química Biológica (Facultad de Ciencias), Universidad de la República, Montevideo, Uruguay; b Faculty of Biology, Medicine and Health, School of Biological Sciences, University of Manchester, Manchester, United Kingdom; University of Pennsylvania

**Keywords:** *Echinococcus*, laminated layer, alum, NLRP3, PI3K, membrane affinity-triggered signaling, adjuvants, dendritic cells, exophagy, helminths, inflammation, macrophages, mucin

## Abstract

The interaction of dendritic cells and macrophages with a variety of rigid noncellular particles triggers activation of the NLRP3 inflammasome and consequent secretion of interleukin 1β (IL-1β). Noncellular particles can also be generated in the context of helminth infection, since these large pathogens often shed their outermost structures during growth and/or molting. One such structure is the massive, mucin-based, soft, flexible laminated layer (LL), which protects the larval stages of cestodes of the genus *Echinococcus*. We show that particles from the Echinococcus granulosus LL (pLL) trigger NLRP3- and caspase-1-dependent IL-1β in lipopolysaccharide (LPS)-primed mouse bone marrow-derived dendritic cells (BMDC). This response can be elicited by pLL too large for phagocytosis and nonetheless requires actin dynamics, Syk, and phosphatidylinositol 3-kinase (PI3K). These three requirements had already been observed in our previous study on the alteration by pLL of CD86, CD40, IL-10, and IL-12 responses to LPS in BMDC; however, we now show that these alterations are independent of NLRP3 and caspase-1. In other words, an initial interaction with particles requiring actin dynamics, Syk, and PI3K, but not phagocytosis, elicits both NLRP3-dependent and NLRP3-independent responses. Intraperitoneal injection of pLL induced IL-1β, suggesting that contact with LL materials induces IL-1β in the E. granulosus infection setting. Our results extend our understanding of NLRP3 inflammasome activation by noncellular particulate materials both to helminth-derived materials and to flexible/soft materials.

## Abstract (precis)

The interaction of dendritic cells and macrophages with a variety of rigid noncellular particles triggers activation of the NLRP3 inflammasome and consequent secretion of interleukin 1β (IL-1β). Noncellular particles can also be generated in the context of helminth infection, since these large pathogens often shed their outermost structures during growth and/or molting. One such structure is the massive, mucin-based, soft, flexible laminated layer (LL), which protects the larval stages of cestodes of the genus *Echinococcus*.

## INTRODUCTION

The NLRP3 inflammasome is an innate immune sensor-response module triggered by an exceptionally wide range of stimuli (1, 2). Recent work suggests that it plays important roles in helminth infections, antagonizing type 2 responses and potentiating Th17/inflammatory responses, with impacts on parasite burden and pathology (3–8). In addition, and counterintuitively, NLRP3 can promote type 2 responses, independently of the inflammasome (9, 10).

The possible triggers for NLRP3 inflammasome activation in helminth infections include endogenous signals generated by inflammation and tissue damage, as well as helminth products themselves (8). To date, all the helminth products shown to trigger the NLRP3 inflammasome are either soluble or exosomal (3, 4, 7, 11). Outside of the field of helminth infection, rigid particulate matter, specifically of a crystalline nature, is well known to activate the NLRP3 inflammasome in macrophages and dendritic cells (DC) previously stimulated (primed) with Toll-like receptor (TLR) agonists (1, 2, 12–24). Helminths, as a result of the turnover of their outermost structures, have great potential for generating insoluble matter within host tissues. However, such matter is physically very different from the crystalline materials known to activate the NLRP3 inflammasome; in fact, the possibility of NLRP3 inflammasome activation by insoluble helminth materials has not been analyzed. One such outermost helminth structure is the laminated layer (LL), a millimeter-thick mucin-based protective structure of the Echinococcus granulosus *sensu lato* larva (25–27). This bladder-like larva causes cystic echinococcosis in livestock and humans (28–30). Larval growth is accompanied by the shedding of LL particles, observed in experimental infections with E. granulosus *sensu lato* (31) and better documented for the closely related species Echinococcus multilocularis (32–34).

We have previously analyzed the immunological effects of a preparation of particles from the E. granulosus *sensu lato* LL (termed pLL) as a possible model of LL particles shed *in vivo* (35–37). pLL are made up of an aqueous gel and are soft and deformable (35). In mouse bone marrow-derived dendritic cells (BMDC), in particular, pLL induce the expression of CD86 and enhance lipopolysaccharide (LPS)-elicited CD86 and interleukin 10 (IL-10) expression while blunting the responses of CD40 and IL-12p70 (as well as its subunit IL-12/23p40) to LPS (35). These changes elicited by pLL require actin dynamics, phosphatidylinositol 3-kinase (PI3K) class I, and probably the kinase Syk, but not particle phagocytosis, and appear to be receptor independent (36). These features match a mechanism termed “membrane affinity-triggered signaling” (MATS), proposed by Yan Shi to explain DC and macrophage responses to solid, mostly crystalline materials (38). In this proposed mechanism, solid surfaces interact with polar headgroups of certain plasma membrane lipids, causing the coalescence of lipid rafts and/or specifically the aggregation of phosphatidylinositol 4,5-bisphosphate (PIP2) (38–41). The cytosolic protein moesin is then recruited to clustered PIP2 and, in turn, causes activation of Syk and downstream signaling that does not require conventional receptors (41). MATS may trigger phagocytosis, but it can operate from the cell surface in the absence of particle internalization (39–41). Materials proposed to act on DC via MATS include sodium urate and alum (39–41), which are additionally known to activate the NLRP3 inflammasome (13, 15).

The mechanistic similarities between responses to pLL and those induced by MATS led us to hypothesize that pLL could also activate the NLPR3 inflammasome. We also wondered whether such activation underlies the changes caused by pLL to BMDC responses to LPS (35, 36). In this paper, we show that pLL do elicit NLRP3-dependent IL-1β from BMDC but that the previously described alterations in BMDC responses to LPS are NLRP3 independent. We also show that NLRP3 inflammasome activation by pLL shares MATS-like requirements with the previously described alterations to LPS responses, adding weight to the hypothesis that DC recognition of pLL involves a MATS-like interaction. Our results extend the range of particulate NLRP3 inflammasome activators to soft/flexible materials and suggest that additional insoluble materials shed by helminths may activate the hosts’ NLRP3 inflammasome module.

## RESULTS

### pLL trigger NLRP3- and caspase-1-dependent IL-1β and IL-18 secretion in primed BMDC.

**FIG 1:**
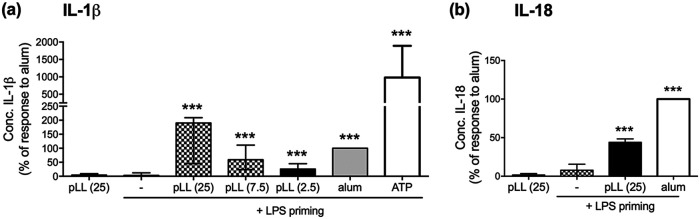
pLL elicit IL-1β and IL-18 production in LPS-primed BMDC. BMDC were either primed with LPS (10 ng/ml) for 2 h or incubated in medium only and were then incubated for a further 3 h with either medium only (–), pLL (at the indicated doses, given in micrograms [dry mass] per million cells), alum (50 μg per million cells), or ATP (2 mM). IL-1β (a) and IL-18 (b) were measured in cell supernatants. Graphs show medians and ranges of results from three (a) or two (b) independent experiments with internal triplicates. Data were normalized to the corresponding responses to alum. The absolute median IL-1β response to alum in primed cells was 11 ng/ml (range, 4 to 27 ng/ml). The absolute median IL-1β responses to pLL in primed cells were 12 ng/ml (range, 8 to 22 ng/ml) for a 25-μg dose, 6 ng/ml (range, 2 to 13 ng/ml) for a 7.5-μg dose, and 3 ng/ml (range, 1 to 5 ng/ml) for a 2.5-μg dose. The absolute median IL-1β response to pLL (25 μg) in nonprimed cells was 0.4 ng/ml (range, 0.2 to 0.4 ng/ml). The absolute median IL-18 response to alum in primed cells was 120 pg/ml (range, 86 to 154 pg/ml), and the corresponding value for a 25-μg dose of pLL was 54 pg/ml (range, 34 to 75 pg/ml). The absolute median IL-18 response to pLL in nonprimed cells was 3 pg/ml (range, 0 to 5 pg/ml). Asterisks represent significant differences (***, *P* < 0.001) from the LPS-only condition (with no second signal).

DC and macrophages that have been primed with TLR agonists release IL-1β and IL-18 upon subsequent exposure to alum or other crystalline materials (1, 2). To find out if pLL could similarly trigger IL-1β and IL-18 release, we exposed LPS-primed BMDC to pLL, or to alum for comparison purposes. pLL induced IL-1β and IL-18 secretion at levels within the same order of magnitude as those elicited by alum (Fig. 1a and b). Either insoluble stimulus induced much less IL-1β than ATP (2 mM), a very potent soluble activator of the NLRP3 inflammasome that acts via the P2X7 receptor (1, 2). Negligible amounts of IL-1β or IL-18 were produced in the absence of LPS priming (Fig. 1a and b), in accord with the previous conclusion that pLL do not contain TLR agonists and/or activate NF-κB (35, 36). Because NLRP3 inflammasome activation is often accompanied by some degree of cell death (1, 2), we measured cell viability following exposure to pLL or alum. Exposure to pLL (at the highest dose used) or alum under the assay conditions caused cell viability to drop from ca. 85% in cells only primed with LPS to ca. 60% (see Fig. S1 in the supplemental material).

In spite of the similar responses to pLL and alum observed in BMDC, a major difference between the two materials was observed upon the stimulation of primed bone marrow-derived macrophages (BMDM). Whereas alum robustly stimulated IL-1β in macrophages also, the impact of pLL appeared to be restricted to BMDC, with only a low-level response in macrophages (Fig. S2).

Reduction of disulfides alters the physicochemical properties of the LL and weakens the effects of pLL on LPS-induced signaling, costimulatory molecule responses, and cytokine responses (35, 36, 42). In agreement with these previous findings, pLL subjected to disulfide reduction elicited a diminished IL-1β response in BMDC (Fig. S3).

**FIG 2:**
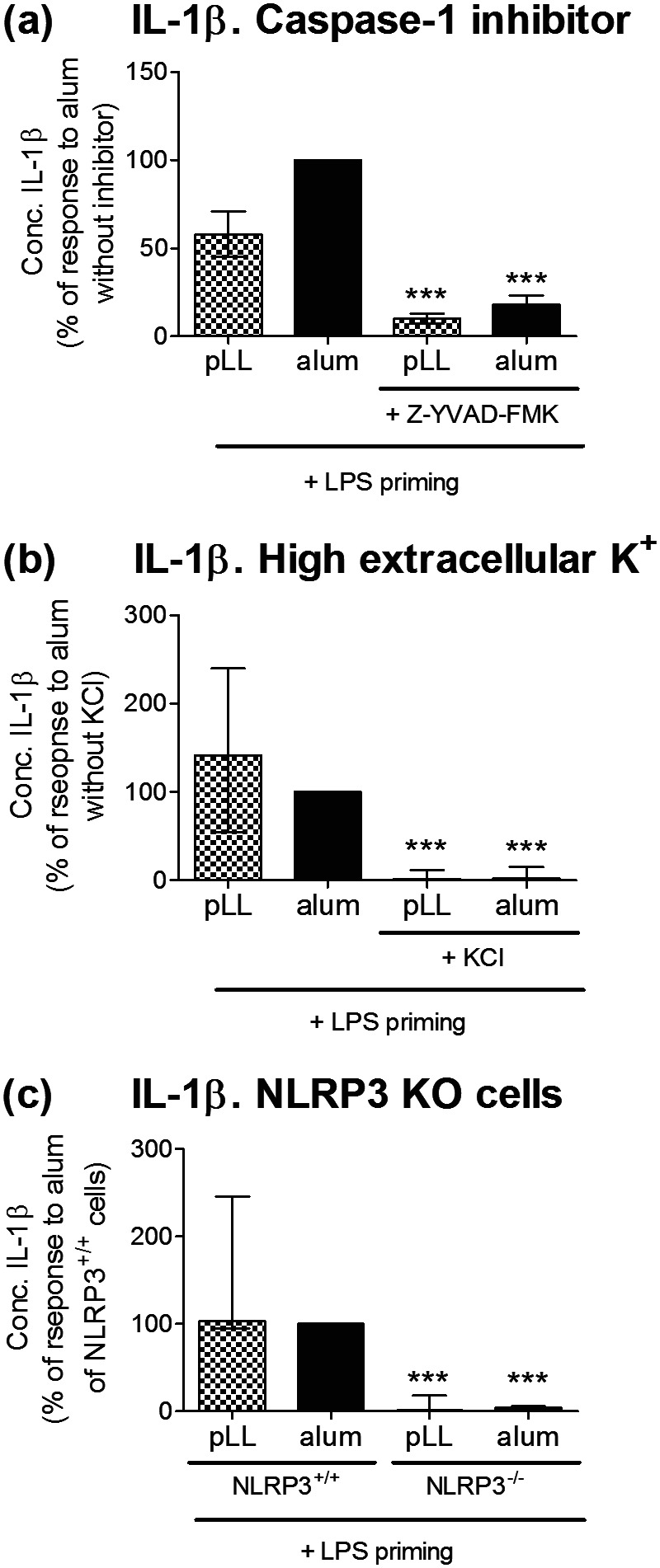
IL-1β production in response to pLL depends on caspase-1 and NLRP3. BMDC were first primed with LPS (10 ng/ml) for 2 h and then incubated for a further 3 h with either medium only, pLL (25 μg [dry mass] per million cells), or alum (50 μg per million cells). IL-1β was measured in cell supernatants. (a and b) Thirty minutes before the addition of pLL or alum, cells were exposed either to the caspase-1 inhibitor Z-YVAD-FMK (a) or to 45 mM KCl (b). (c) Alternatively, cells from NLRP3^+/+^ and NLRP3^−/−^ mice were compared. Graphs show medians and ranges of results from two (a), four (b), or three (c) independent experiments with internal triplicates. Data were normalized to the corresponding responses to alum in the absence of inhibitors (a and b) or in NLRP3^+/+^ animals (c). The median absolute values of responses to alum were 21 ng/ml (range, 16 to 27 ng/ml) (a), 19 ng/ml (range, 4 to 47 ng/ml) (b), and 14 ng/ml (range, 3 to 34 ng/ml) (c). The median absolute values of responses to pLL in the absence of inhibitors and in wild-type cells were 12 ng/ml (range, 11 to 12 ng/ml) (a), 23 ng/ml (range, 8 to 26 ng/ml) (b), and 14 ng/ml (range, 6 to 33 ng/ml) (c). KO, knockout. Asterisks represent significant differences (***, *P* < 0.001) from the results of stimulation with the particles in the presence of vehicle only (a and b) or from results for NLRP3^+/+^ cells (c).

To determine if IL-1β production by pLL in primed BMDC reflects NLRP3 inflammasome activation, the experiments were repeated in the presence of a caspase-1 inhibitor (Fig. 2a) or 45 mM extracellular K^+^ (Fig. 2b), both known to inhibit NLRP3-dependent IL-1β production (1). With both treatments, IL-1β production induced by pLL was strongly inhibited (Fig. 2a and b). Moreover, no induction of IL-1β secretion by pLL was observed in primed BMDC deficient in NLRP3 (Fig. 2c). The results for pLL matched the results obtained for alum (Fig. 2a to c).

In sum, these data provide strong evidence that pLL activate the NLRP3 inflammasome in primed BMDC.

### pLL triggers both NLRP3- and caspase 1-dependent and -independent responses.

**FIG 3:**
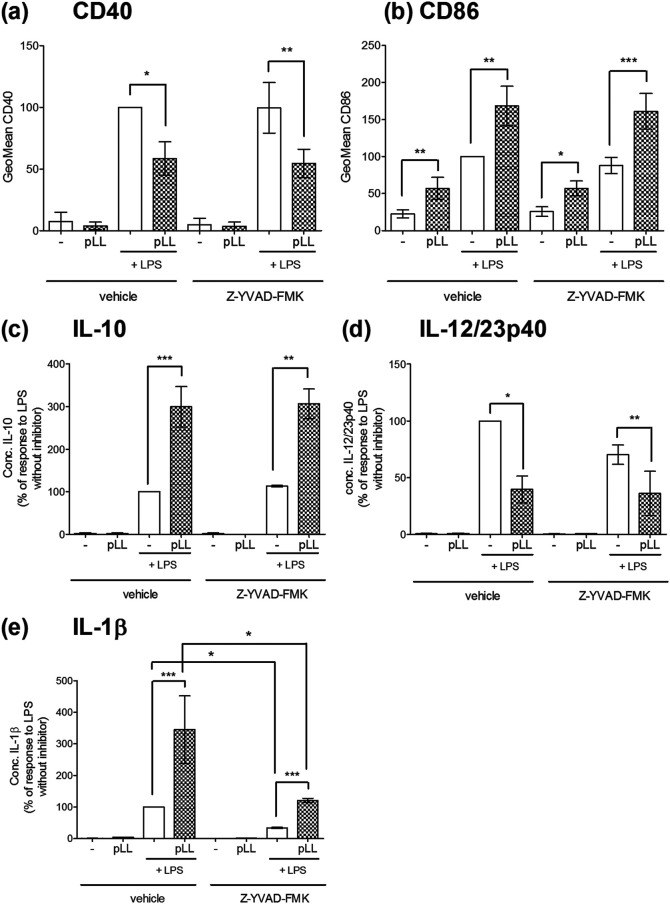
pLL induce caspase 1-independent responses in BMDC in parallel to eliciting caspase-1-dependent IL-1β. BMDC were exposed to pLL (25 μg [dry mass] per million cells) or medium only and were stimulated 1 h later with LPS (10 ng/ml) or added medium only; the experiment was carried out in the presence or absence of the caspase-1 inhibitor Z-YVAD-FMK. Eighteen hours later, the expression of CD40 (a) and CD86 (b) at the cell surface and the expression of IL-10 (c), IL-12/23p40 (d), and IL-1β (e) in supernatants were measured. Graphs show medians and ranges of results from two independent experiments with internal duplicates. Data were normalized to the corresponding responses to LPS as the sole stimulus. The median absolute values of responses to LPS alone were 0.4 ng/ml (range, 0.2 to 0.6 ng/ml) for IL-10, 5 μg/ml (range, 0.4 to 9 μg/ml) for IL-12/23p40, and 6 ng/ml (range, 5 to 7 ng/ml) for IL-1β. The median absolute values of responses to pLL plus LPS in the absence of inhibitor were 1.3 ng/ml (range, 0.5 to 2.1 ng/ml) for IL-10, 1.4 μg/ml (range, 0.2 to 2.6 μg/ml) for IL-12/23p40, and 20 ng/ml (range, 17 to 23 ng/ml) for IL-1β. *, *P* < 0.05; **, *P* < 0.01; ***, *P* < 0.001. Note that only in terms of IL-1β output were significant differences detected between conditions in the presence of the inhibitor and those in its absence.

**FIG 4:**
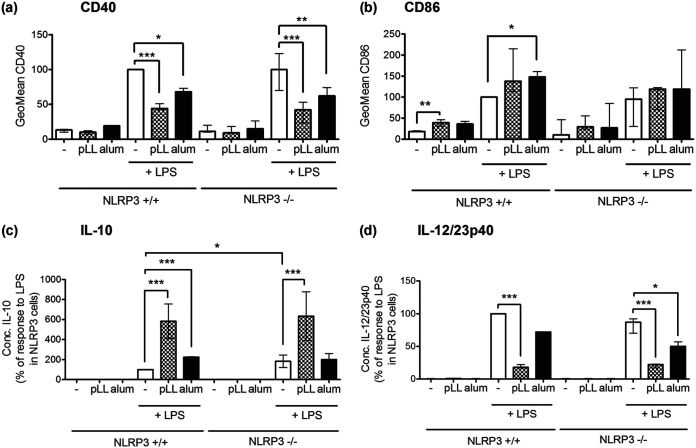
pLL and alum both elicit NLRP3-independent cellular responses. BMDC from either NLRP3^+/+^ or NLRP3^−/−^ mice were exposed to either pLL (25 μg [dry mass] per million cells), alum (25 μg per million cells), or medium only and were stimulated 1 h later with LPS (10 ng/ml) or added medium only. Eighteen hours later, the expression of CD40 (a) and CD86 (b) at the cell surface was measured by flow cytometry, and IL-10 (c) and IL-12/23p40 (d) in supernatants were quantitated by ELISA. Graphs show medians and ranges of results from three independent experiments with internal triplicates. Data were normalized to the corresponding responses of NLRP3^+/+^ cells to LPS as the sole stimulus. The median absolute values of responses to LPS alone in wild-type cells were 1.3 ng/ml (range, 0.9 to 1.7 ng/ml) for IL-10 and 0.6 μg/ml (range, 0.3 to 0.9 g/ml) for IL-12/23p40. *, *P* < 0.05; **, *P* < 0.01; ***, *P* < 0.001. Note that no significant differences were detected between conditions in the presence of the inhibitor and those in its absence.

Exposure to pLL alters BMDC responses to LPS: it enhances CD86 expression and IL-10 secretion, whereas it blunts CD40 expression and IL-12/23p40 secretion, as reported previously (35, 36) and confirmed here (Fig. 3a to d). We wondered if these alterations were a consequence of pLL-mediated activation of NLRP3 inflammation. In the 18-h format of these experiments (see Materials and Methods) and in agreement with previous data (43), LPS by itself triggered an IL-1β response, but this response was much potentiated by the addition of pLL (Fig. 3e). In the absence of LPS, pLL induced negligible IL-1β. The response to LPS plus pLL (as well as the response to LPS alone) was reduced by the caspase-1 inhibitor (Fig. 3e). Therefore, LPS can act as a priming stimulus, and pLL as a second signal, in an assay format that is not designed to elicit/measure NLRP3 inflammasome activation. In contrast to the findings for IL-1β, the effects of pLL on the LPS-driven expression of CD86, CD40, IL-10, and IL-12/23p40 were completely unaltered by chemical inhibition of caspase-1 (Fig. 3a to d). Moreover, the effects of pLL on CD86, CD40, IL-10, and IL-12/23p40 were also independent of NLRP3 in the same assay format (Fig. 4a to d).

Of note, and in agreement with previous reports (39, 44, 45), alum also inhibited CD40 upregulation and IL-12/23p40 secretion and potentiated IL-10 secretion in the context of LPS stimulation (Fig. 4a, c, and d); these effects were also independent of NLRP3 (Fig. 4a, c, and d).

In sum, the effects of pLL on the LPS-induced changes in CD40, CD86, IL-10, and IL-12/23p40 are independent of NLRP3 inflammasome activation.

### The IL-1β responses induced by pLL require actin dynamics, Syk, and PI3K signaling.

**FIG 5:**
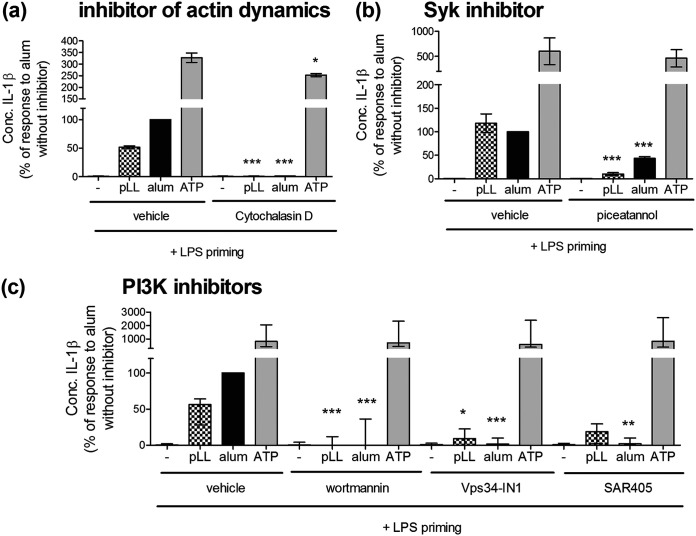
Induction of IL-1β production by pLL or alum requires components of the phagocytic machinery. BMDC were first primed with LPS (10 ng/ml) for 2 h and then incubated for a further 3 h with either medium only, pLL (25 μg [dry mass] per million cells), alum (50 μg per million cells), or ATP (2 mM), and IL-1β was measured in supernatants. Thirty minutes before the second signal, cells were exposed to inhibitors of actin dynamics (a), Syk (b), or PI3K enzymes (c) or vehicle (DMSO) only. Graphs show medians and ranges of results from two (a and b) or three (c) independent experiments with internal triplicates or duplicates. Data were normalized to the corresponding responses to alum in the absence of inhibitors. The median absolute values of responses to alum in the absence of inhibitors were 39 ng/ml (range, 32 to 45 ng/ml) (a), 19 ng/ml (range, 17 to 22 ng/ml), and 8 ng/ml (range, 5 to 11 ng/ml). The median absolute values of responses to pLL in the absence of inhibitors were 20 ng/ml (range, 17 to 23 ng/ml) (a), 23 ng/ml (range, 21 to 24 ng/ml) (b), and 3 ng/ml (range, 3 to 7 ng/ml) (c). Asterisks represent significant differences from results for the corresponding conditions in the absence of inhibitors. *, *P* < 0.05; **, *P* < 0.01; ***, *P* < 0.001.

As mentioned previously, the effects of pLL on the LPS-induced CD86, CD40, IL-10, and IL-12 responses in BMDC are abrogated by inhibitors of actin dynamics or PI3K class I and are also affected by Syk inhibitors (35, 36). Also, NLRP3-dependent responses to particulate stimuli have been reported to depend on actin dynamics, PI3K, and/or Syk (17–21, 44). Thus, we wondered whether the NLRP3-dependent responses to pLL shared these mechanistic requirements. Indeed, IL-1β production elicited by pLL was strongly inhibited by blockade of actin polymerization (cytochalasin D) (Fig. 5a) or Syk signaling (piceatannol) (Fig. 5b) or by a pan-PI3K inhibitor (wortmannin) (Fig. 5c). Moreover, inhibition of either PI3K class I (Fig. S4) or PI3K class III (Fig. 5c) individually led to similar reductions in IL-1β release. Whereas IL-1β secretion in response to alum displayed similar requirements, the corresponding response to the nonparticulate stimulus ATP was unaffected or only weakly inhibited (Fig. 5a to c).

We reported previously that exposure of BMDC to pLL blunts the activation of the PI3K class I effector Akt elicited by disparate agonists (LPS, granulocyte-macrophage colony-stimulating factor [GM-CSF], IL-4) (36, 37). This is a somewhat paradoxical effect in view of the overarching requirement for PI3K class I activity in order for pLL to affect BMDC responses (Fig. 5c; Fig. S4) (36). Interestingly, alum did not share the ability of pLL to blunt Akt phosphorylation in response to LPS (Fig. S5), suggesting that BMDC responses to pLL and alum have mechanistic differences and that the effect of pLL on Akt is not a direct consequence of the participation of PI3K in the overall effects of the particles.

In sum, induction of NLRP3-dependent IL-1β production by pLL required several elements of the phagocytic machinery, and these requirements were shared by the particulate stimulus alum but not by the soluble stimulus ATP.

### Laminated-layer particles induce IL-1β in the absence of particle phagocytosis.

**FIG 6:**
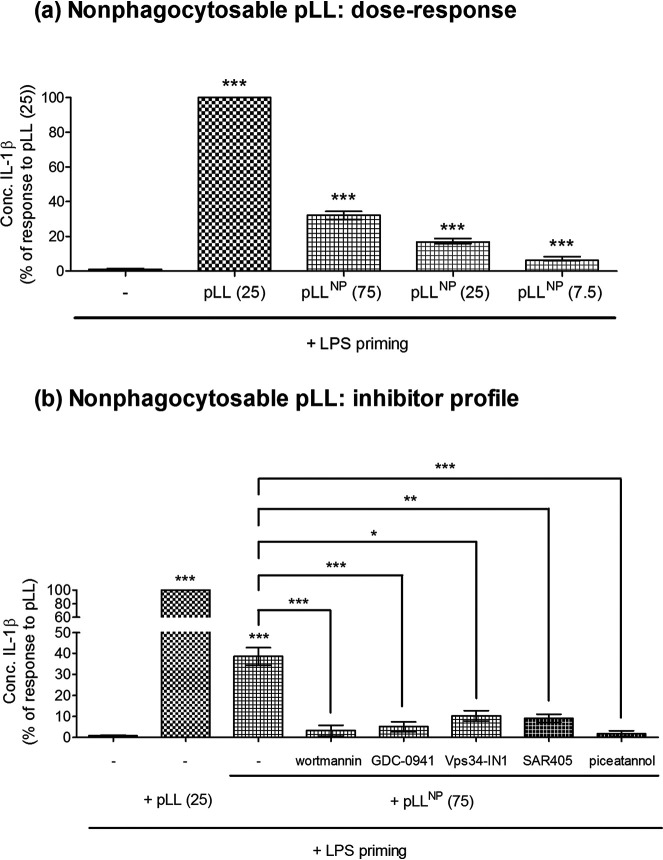
IL-1β production can be induced by pLL in the absence of phagocytosis. BMDC were first primed with LPS (10 ng/ml) for 2 h and then incubated for a further 3 h with either medium only, pLL, or pLL^NP^ (at the indicated doses, expressed in micrograms [dry mass] per million cells), and IL-1β was measured in supernatants. (a) Different doses of pLL^NP^ were assayed in parallel with pLL. (b) Cells were exposed (30 min before pLL^NP^) to the pan-PI3K inhibitor wortmannin, the PI3K class I-specific inhibitor GDC-0941, the PI3K class III-specific inhibitor Vps34-IN1 or SAR405, or the Syk inhibitor piceatannol. Graphs show medians and ranges of results from three (a) or two (b) independent experiments with internal triplicates. Data were normalized to responses to pLL (25 μg). Median absolute responses to pLL (25 μg) and pLL^NP^ (75 μg) in the absence of inhibitors were 16 ng/ml (range, 8 to 22 ng/ml) and 5 ng/ml (range, 2 to 8 ng/ml), respectively. Asterisks not associated with connecting lines represent significant differences from cells stimulated with LPS only. *, *P* < 0.05; **, *P* < 0.01; ***, *P* < 0.001.

Based on existing knowledge, one would assume that the requirements of actin dynamics, Syk signaling, and PI3K signaling for pLL to induce IL-1β are due to a need for pLL to be internalized in order to trigger NLRP3 inflammasome activation. Indeed, particulate NLRP3 inflammasome activators are generally thought to act via phago(lyso)somal destabilization, which requires prior internalization (1, 22, 23, 46). To date, however, we have been unable to detect successful phagocytosis of pLL by BMDC (unpublished observations). Further, a pLL preparation in which all particles are too large for phagocytosis (nonphagocytosable pLL [pLL^NP^]), which we previously showed to have effects on IL-10, IL-12, CD86, and CD40 similar to those of pLL (36), also elicited a clear IL-1β response (Fig. 6a). This response was quantitatively weaker than the response to pLL at the same dry mass dose; this is expected if the effects of pLL originate at the cell surface, since at equal masses, pLL^NP^ have less total surface area than pLL. Moreover, the response to pLL^NP^, like the response to pLL, was sensitive to PI3K class I, PI3K class III, Syk, and actin polymerization inhibitors (Fig. 6b; Fig. S6), suggesting that the same mechanisms are operative in both cases.

Thus, interaction with LL particles at the cell surface can trigger NLRP3-dependent responses, which nonetheless require elements of the phagocytic machinery.

### pLL induces IL-1β *in vivo*.

**FIG 7:**
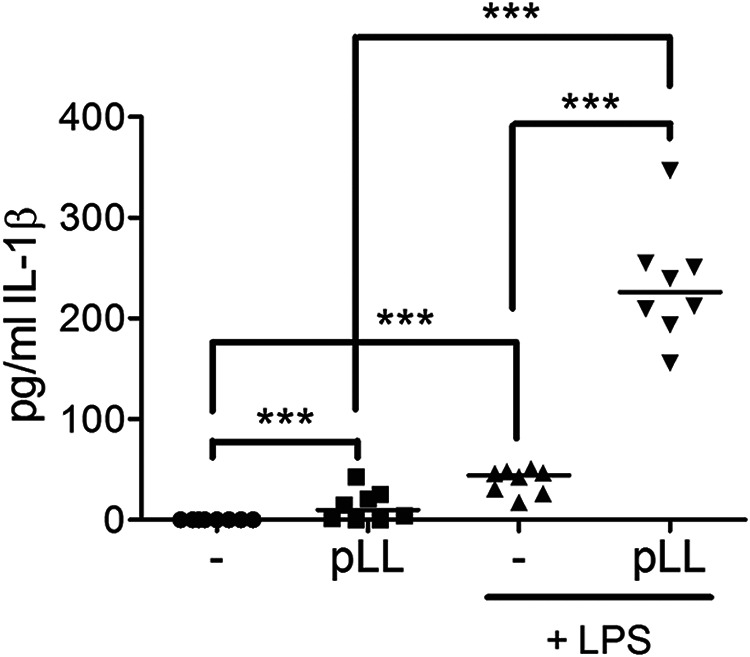
pLL elicits IL-1β *in vivo*. C57BL/6 mice were injected i.p. with either vehicle alone (PBS), pLL (150 μg [dry mass] per mouse), LPS (15 μg per mouse), or pLL plus LPS. Three hours later, IL-1β was measured in the peritoneal lavage fluid. The graph shows medians and data for individual mice pooled from two independent experiments (*n*, 3 and 5). Statistical significance was estimated by a two-way method (specified in Materials and Methods) in which the fact that the data arise from two separate experiments is taken into account. ***, *P* < 0.001.

To determine whether the NLRP3 inflammasome may be activated by particles from the LL of E. granulosus *in vivo*, pLL were injected intraperitoneally (i.p.) into C57BL/6 mice with or without coinjection of LPS (Fig. 7). As expected, LPS instillation induced detectable IL-1β in the lavage fluids of treated animals. In line with our *in vitro* data, pLL drastically enhanced the release of IL-1β, confirming the ability of pLL to act as an NLRP3 inflammasome trigger. In the absence of LPS coinjection, pLL induced modest but significant levels of IL-1β, in line with *in vivo* results with known particulate NLRP3 inflammasome activators (14).

## DISCUSSION

In this work, we report that a noncellular insoluble helminth-derived material (pLL) elicits NLRP3 inflammasome-dependent cytokine production in primed BMDC by a mechanism requiring the phagocytic machinery but not particle internalization. Together with our previous results (36), the observations made in this work suggest that all the responses elicited by pLL in BMDC start with a MATS-like cell surface interaction. This is in contrast to findings for Schistosoma mansoni soluble egg antigen, which activates the NLRP3 inflammasome by a mechanism requiring the receptor dectin-2 and is insensitive to inhibition of actin dynamics (3).

Downstream of the proposed MATS-like cell surface interaction, pLL elicit responses that are both NLRP3/caspase-1 dependent and independent (Fig. 2 and 4). Parallel NLRP3/caspase-1–dependent and –independent responses in DC or macrophages have also been observed for crystalline particulate adjuvants (14, 47) (results for alum in the present work [Fig. 4]). This suggests that eliciting both NLRP3-dependent and -independent responses may be a general feature of materials that trigger MATS-like signaling, independent of the type of material. However, some mechanistic features must differ with the material eliciting the signaling, as suggested by the ability of pLL, but not alum, to inhibit Akt activation (Fig. S5). We previously proposed a tentative mechanism to explain the paradoxical effect of pLL on Akt activation, based on competition for the PI3K class I substrate phosphatidylinositol 4,5-bisphosphate between the synapse with pLL and conventional PI3K-coupled receptors (36). If this mechanism is correct, it may not be operative in the synapse with alum, due to qualitative, or perhaps quantitative, differences from the synapse formed with pLL.

The IL-1β response to pLL was abrogated by PI3K class III inhibitors (Fig. 5 and 6). Explanation of this observation is made difficult by the major uncertainties currently surrounding the mechanisms of NLRP3 inflammasome activation (23, 48). However, since our results suggest that PI3K class III is needed for NLRP3 inflammasome activation in response to pLL/pLL^NP^ and alum, but not for activation in response to ATP (Fig. 5 and 6), the possibility that this enzyme’s role is fulfilled at the synapse with particles should be considered. Neither pLL nor alum (39) appears to be internalized by DC. Particles that cannot be phagocytosed trigger “digestive exophagy,” in which the extracellular equivalent of a lysosome is formed at the synapse with the particles (49), and PI3K class III is needed for (conventional) phagolysosome biogenesis (50). Exophagy has been observed in particular with the cell model used in our work (BMDC generated in the presence of GM-CSF), in response to aggregated low-density lipoprotein (LDL), a probable NLRP3 inflammasome trigger (51, 52). Therefore, we reason that elements of digestive exophagy may be required for NLRP3 inflammasome activation by pLL and alum (and perhaps further particulates); this would explain the requirement for PI3K class III. In contrast, digestive exophagy would not be needed for the NLRP3-independent responses to particulates that also take place in parallel, as suggested by our previous observation that the effects of pLL on LPS-elicited CD86, CD40, IL-10, and IL-12 do not require PI3K class III (36). Integrating the ideas discussed above, we propose that an interaction with MATS-like requirements in the context of frustrated phagocytosis would trigger two lines of signaling: (i) PI3K class III-dependent (possibly exophagy-dependent) signaling leading to NLRP3 inflammasome activation and (ii) PI3K class III-independent signaling leading to the changes observed in other LPS-initiated responses. This process is summarized in Fig. S7 in the supplemental material.

In addition to the mucin-based aqueous gel, the E. granulosus LL contains nanodeposits of calcium inositol hexakisphosphate (InsP_6_) (53–55). CD86, CD40, IL-12, and IL-10 responses to LL materials in BMDC are not appreciably affected by the presence or absence of this component (35). *In vitro* IL-1β production by BMDC was also not appreciably affected by the presence or absence of calcium InsP_6_ (data not shown). Thus, the LL mucin-based gel appears to be sufficient to trigger the proposed MATS-like interaction with the BMDC surface.

As mentioned above, the insoluble materials so far reported to activate the NLRP3 inflammasome are crystalline or otherwise rigid. Accordingly, NLRP3 inflammasome activation by these materials is generally accepted to be triggered by phago(lyso)somal disruption after particle phagocytosis (1, 22, 23, 46). However, some authors have demonstrated NLRP3 inflammasome activation by crystals immobilized on plastic or by particles too large for phagocytosis (56, 57). These authors propose that a MATS-like cell surface interaction may also trigger NLRP3 inflammasome activation. Our results support this proposal and, importantly, extend NLRP3 inflammasome activation to a material that is an aqueous gel, and hence soft. Material-level features of soft noncellular materials probably determine their potential to activate the NLRP3 inflammasome. This is suggested by our observation that the reduction of disulfides in pLL weakens its capacity to elicit an IL-1β response (Fig. S3). Disulfide reduction facilitates the solubilization of the LL mucin meshwork upon sonication (42). We envisage that disulfide reduction alters the physical properties of pLL so that they fall below a minimum level of stiffness required for the MATS-like interaction.

The *in vivo* IL-1β response elicited by pLL was much enhanced by LPS, but it nonetheless reached statistically significant levels in the absence of LPS coinjection (Fig. 7). This is consistent with previous observations suggesting that priming signals for myeloid cell responses to particulate adjuvants are ubiquitous *in vivo* (14). Therefore, in the E. granulosus infection setting, DC in contact with shed LL particles probably generate IL-1β (and IL-18). In addition, and as suggested by our observations with nonphagocytosable particles, cells in contact with the surface of the LL as such may also respond with IL-1β. From precedents in other helminth infections, IL-1β (and IL-18) could contribute to local inflammation, downregulate Th2 responses, and/or promote Th1 and Th17 responses, both of which are detectable in cystic echinococcosis (28, 58–60). Shed particles from the E. multilocularis LL (32–34) may also be NLRP3 inflammasome triggers and may contribute to the considerable inflammatory response observed in human patients (61).

Since the survival strategy of larval E. granulosus is based on inflammatory control (25, 28, 61), we hypothesize that the parasite may have evolved means to curtail NLRP3 activation. A major secreted E. granulosus lipoprotein, “antigen B” (not contained in pLL), inhibits IL-1β output in THP-1 macrophages (62), although it is not yet clear whether this effect involves inhibition of NLRP3 activation.

pLL are the first biological particulate material to be shown to activate the NLRP3 inflammasome independently of phagocytosis. Other noncellular surface structures shed by tissue-dwelling helminths may share this potential. In particular, it is conceivable that nematode cuticles shed during molting may be NLRP3 inflammasome triggers and thus contribute to local inflammation in response to these helminths.

## MATERIALS AND METHODS

### Parasite materials.

pLL, pLL treated for reduction/alkylation of disulfides, and nonphagocytosable pLL (pLL^NP^) were generated, had their concentrations determined, and were stored as described elsewhere (35, 36). The preparation of pLL involves a dehydration step followed by grinding into a fine powder, rehydration, and filtration of the resulting suspension; of the two dehydration methods described in reference 35, freeze-drying was used in the present work. pLL preparations tested negative for endotoxin by the *Limulus* amebocyte lysate (LAL) method (35).

### BMDC generation and stimulation.

BMDC were generated in the presence of GM-CSF as described elsewhere (35, 36) from female C57BL/6 wild-type or NLRP3 gene-deficient (B6.129S6-Nlrp3^tm1Bhk^/J) mice (The Jackson Laboratory). For inflammasome activation assays, BMDC (400,000 cells per well of 96-well plates) were either primed for 2 h with 10 ng/ml LPS (from Escherichia coli O127:B8; Sigma) or incubated with medium only (final volume in priming step, 100 μl). Then the cells were stimulated with either pLL, pLL^NP^, alum (Alhydrogel; Invivogen), or ATP (Sigma) or were incubated with medium only (additional volume, 100 μl). Supernatants were collected 3 h later. For assays determining effects on LPS-induced costimulatory molecule expression and cytokine output in BMDC, pLL or alum was added (the final volume in this step was 100 μl), followed 1 h later by LPS (10 ng/ml, in 100 μl additional volume), and cells and supernatants were harvested 18 h later (35). Cell viability was measured on the basis of exclusion of the To-Pro-3 (50 nM; Invitrogen) viability probe in flow cytometry.

### Generation and stimulation of BMDM.

Bone marrow-derived macrophages (BMDM) were generated from the bone marrow of female C57BL/6 mice by differentiation for 7 days in the presence of L929 cell supernatant as a source of M-CSF, as described in reference 63. Cells were stimulated for inflammasome activation as described for BMDC, except that they were plated at 200,000 cells per well of 96-well plates, and the doses of pLL and alum used were each 50 μg per million cells.

### Chemical inhibitors.

The following chemical inhibitors were used: Z-YVAD-FMK (10 μM; Calbiochem/Merck), wortmannin (1 μM; Sigma), GDC-0941 (5 μM; Calbiochem/Thermo), piceatannol (25 μM; Santa Cruz Biotechnology), cytochalasin D (5 μM; Sigma-Aldrich), and Vps34-IN1 (1 μM) and SAR405 (1 μM) (both from the Division of Signal Transduction Therapy Unit, University of Dundee, Dundee, Scotland).

### Measurement of cell responses.

Cytokines in supernatants were measured with commercial enzyme-linked immunosorbent assay (ELISA) kits from R&D Systems (DuoSet ELISA kit for IL-1β and IL-10) and eBioscience (IL-18 mouse ELISA kit), or with an antibody pair formed by an unconjugated antibody from BD Pharmingen and a biotinylated antibody from BioLegend (IL-12/23p40). The expression of CD40 and CD86 was measured by flow cytometry in cells gated for CD11c expression, as described in reference 35.

### Measurement of Akt phosphorylation.

BMDC were stimulated with LPS (10 ng/ml) alone or together with pLL or alum for 80 min (36). Phosphorylation of Akt at Ser^473^ was measured by Western blotting as described elsewhere (36, 37).

### *In vivo* effects of pLL.

C57BL/6 mice (female, 8 to 10 weeks old) were injected i.p. with pLL (150 μg [dry mass] per mouse), LPS (15 μg per mouse), both pLL and LPS, or vehicle only (200 μl of phosphate-buffered saline [PBS]). Three hours later, mice were euthanized using isoflurane, and peritoneal lavage fluid was collected for IL-1β quantification.

### Statistics.

Data were analyzed by a nonparametric method, thus avoiding having to assume normality and homogeneity of variances. Specifically, the extension of the Friedman test with the Conover posttest and the Bonferroni correction was applied (64). This method incorporates data corresponding to internal repetitions within each of two or more experiments; it allows for interexperiment variation in the absolute values obtained, and it identifies those differences between conditions that are consistent across experiments. The number of independent experiments and internal repetitions used for statistical analysis and summarized in the graphs shown is given in each figure legend. For graphical presentation purposes, some data were normalized to responses to alum or pLL; however, statistical analyses were always carried out on the crude data. Significances are indicated by asterisks in figures and are explained in the figure legends.

## Supplementary Material

Supplemental file 1
