# Supplementary material for: Activation of the NLRP3 Inflammasome by Particles from the Echinococcus granulosus Laminated Layer
Source: Infect Immun. 2020 Aug 19;88(9):e00190-20. doi: 10.1128/IAI.00190-20 (PMC7440765; doi:10.1128/IAI.00190-20)
Supplement: Supplemental file 1 [file IAI.00190-20-s0001.pdf]

Figure S1

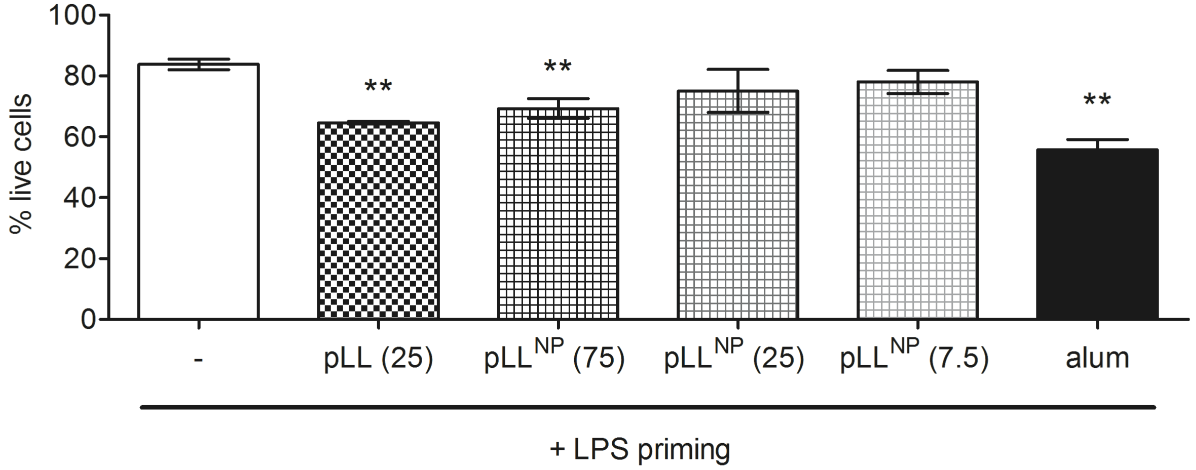

Suppl. Fig. 1. Exposure to pLL or alum under inflammasome assay conditions causes moderate levels of cell death. BMDC were primed with LPS (10 ng/ml) for 2 h, then incubated for a further 3 h with medium only, pLL, pLL^{NP} (at the indicated doses, given in µg dry mass per million cells) or alum (50 µg per million cells). Then, cell viability was measured by flow cytometry using the To-Pro3 probe. The graph shows median and ranges of 2 independent experiments with internal duplicates. Asterisks represent significant differences with respect to the control (no second signal) condition.

Figure S2

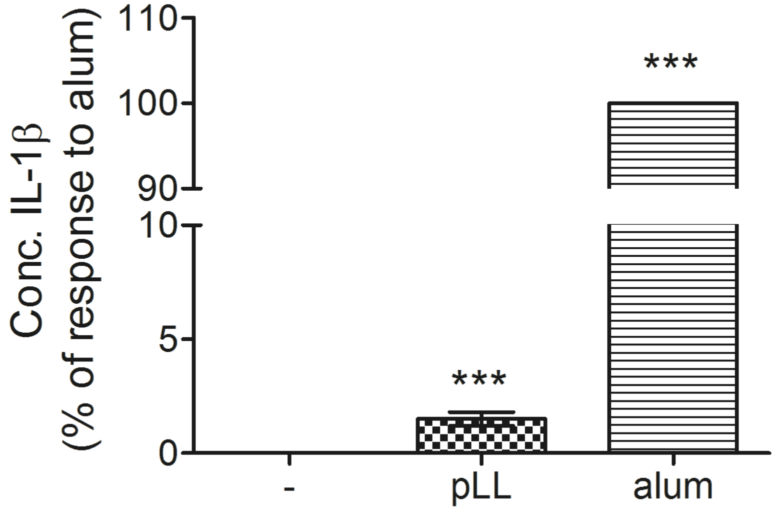

Suppl. Fig. 2. pLL induces only minimal IL-1β production in LPS-primed BMDM. BMDM were primed with LPS (10 ng/ml) for 2 h, then incubated for a further 3 h with medium only, pLL (50 µg dry mass per million cells) or alum (50 µg per million cells), and IL-1β was measured in cell supernatants (IL-18 was not detectable). The graph shows median and ranges of 2 independent experiments with internal triplicates. Values were normalized over the corresponding responses to alum. The median absolute value of this response to alum and pLL were 5 ng/mL (range 3-7 ng/mL) and 0.06 ng/mL (range 0.05-0.07 ng/mL) respectively. Asterisks represent significant differences with respect to the control (no second signal) condition.

Figure S3

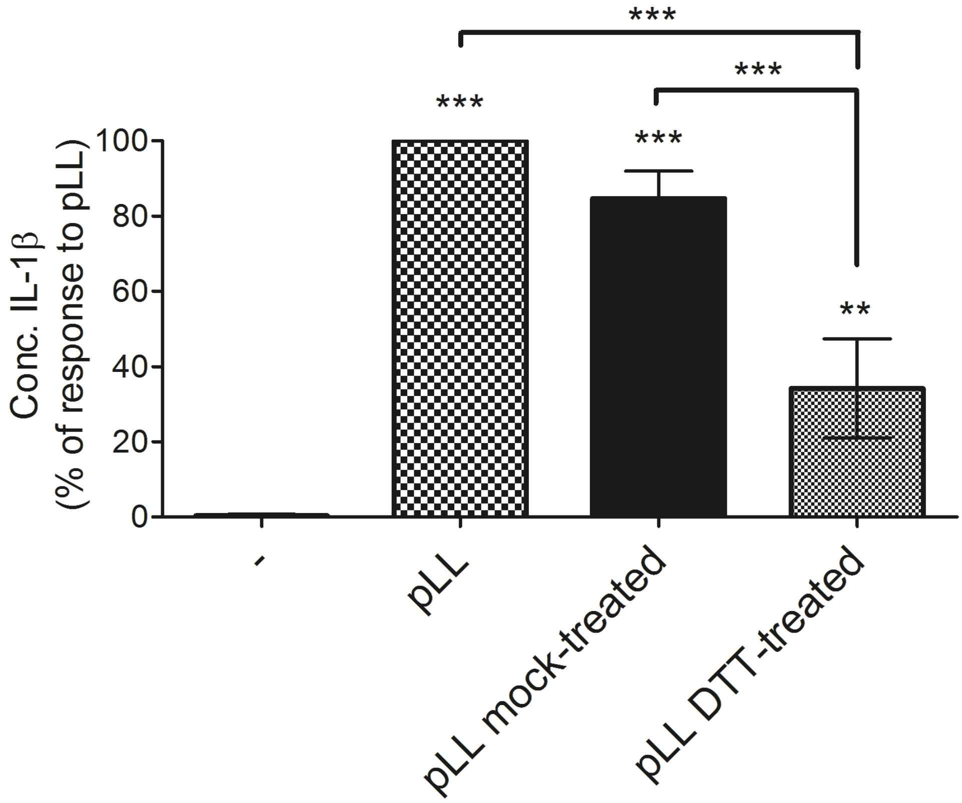

Suppl. Fig. 3. Disulfide reduction weakens the capacity of pLL to induce IL-1β production. BMDC were primed with LPS (10 ng/ml) for 2 h, then incubated for a further 3 h with medium only or pLL (25 µg dry mass per million cells), and IL-1β was measured in cell supernatants. pLL was untreated, subjected to reduction of disulfides using DTT followed by blocking of free thiols generated using iodoacetamide, or subjected to mock treatment (iodoacetamide only). The graph shows median and ranges of 2 independent experiments with internal triplicates. Values were normalized over the corresponding responses to untreated pLL; the median absolute value of this response was 10 ng/mL (range 5-15 ng/mL). Asterisks without connecting lines represent significant differences with respect to the LPS-only (no second signal) condition.

Figure S4

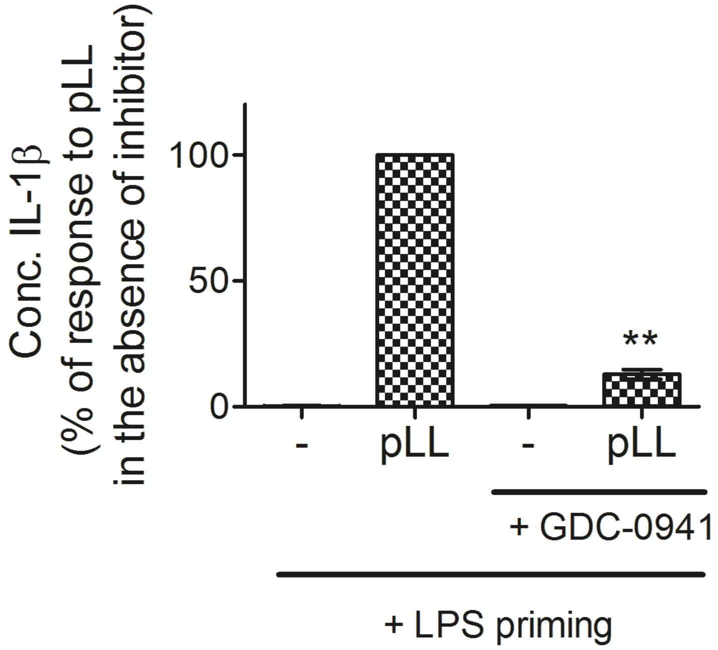

Suppl. Fig. 4 (related to Fig. 5). Induction of IL-1β production by pLL requires PI3K class I. BMDC were primed with LPS (10 ng/ml) for 2 h, then incubated for a further 3 h with medium only or pLL (25 µg dry mass per million cells) and IL-1β was measured in supernatants. Thirty minutes before the second signal, cells were exposed to a selective inhibitor of PI3K class I (GDC-0941) or vehicle only. The graph shows median and ranges of 2 independent experiments with internal duplicates. Values were normalized over the corresponding responses to pLL in the absence of inhibitor; the median absolute value of this response was 9 ng/mL (range 6-13 ng/mL). Asterisks represent significant differences with respect to the corresponding condition without inhibitor.

Figure S5

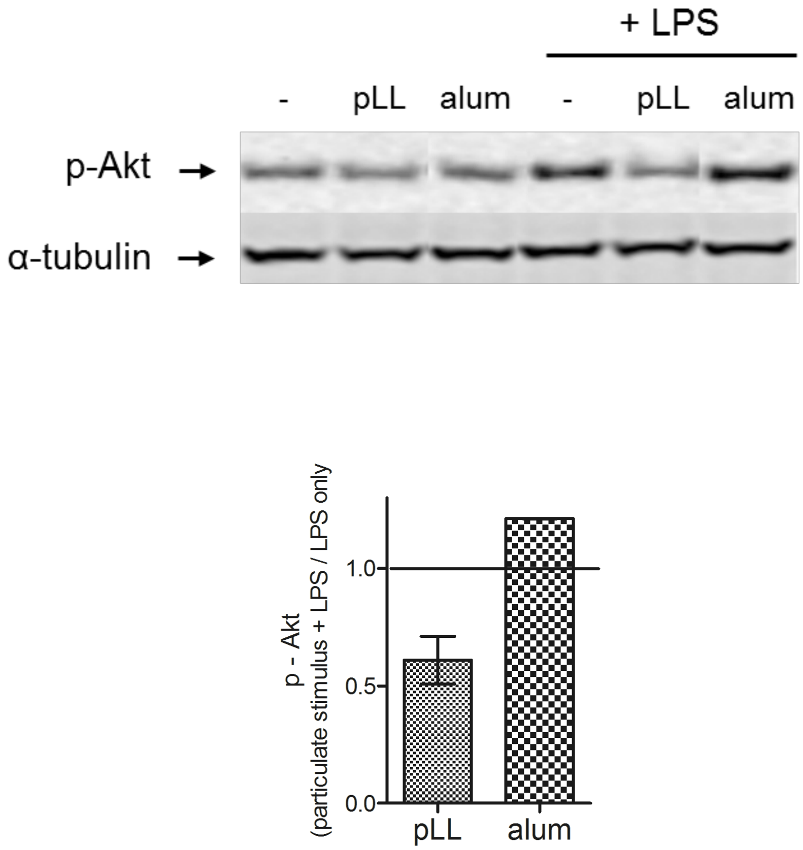

Suppl. Fig. 5. The effects of pLL and alum on LPS-stimulated Akt phosphorylation are different. BMDC were exposed to pLL (25 µg dry mass per million cells), alum (50 µg per million cells) or vehicle only, in the absence or presence of LPS (10 ng/mL). After 80 min, cell lysates were analyzed for Akt phosphorylated at the Ser^{473} position. The Western blot shown is representative of two independent experiments. The graph shows the quotients of p-Akt values (normalized over loading controls) for cells treated with either pLL or alum plus LPS over cells exposed to LPS only (median and range of the 2 experiments).

Figure S6

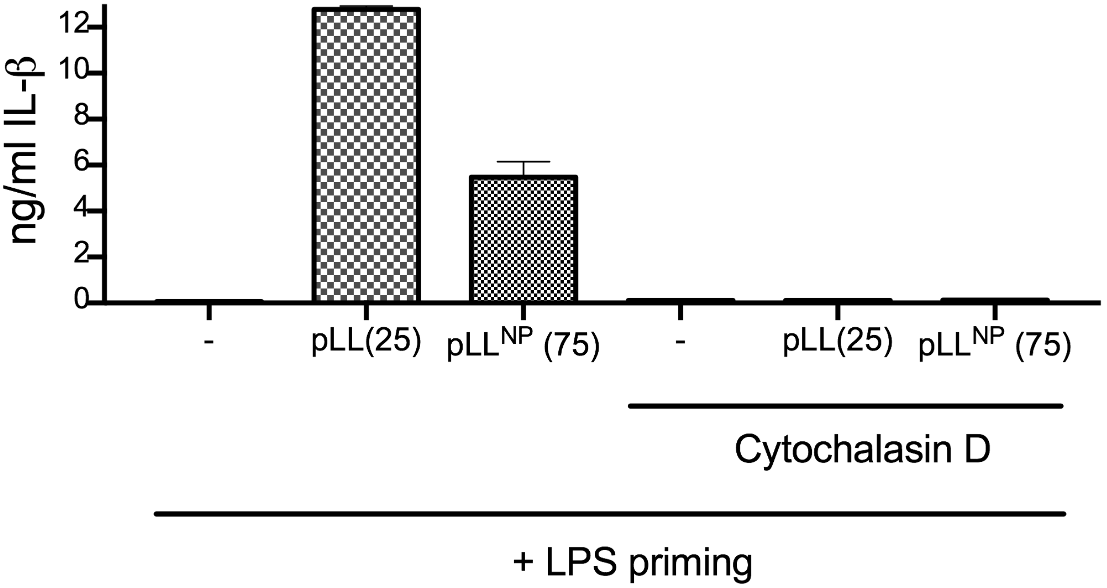

Suppl. Fig. 6 (related to Fig. 6). Induction of IL-1β production by non-phagocytosable pLL is abrogated by an inhibitor of actin dynamics. BMDC were primed with LPS (10 ng/ml) for 2 h, then incubated for a further 3 h with medium only or pLL or pLL^{NP} (75 µg dry mass per million cells) and IL-1β was measured in supernatants. Thirty minutes before the second signal, cells were exposed to Cytochalasin D or vehicle only. The graph shows median and ranges of internal triplicates from a single experiment.

Figure S7

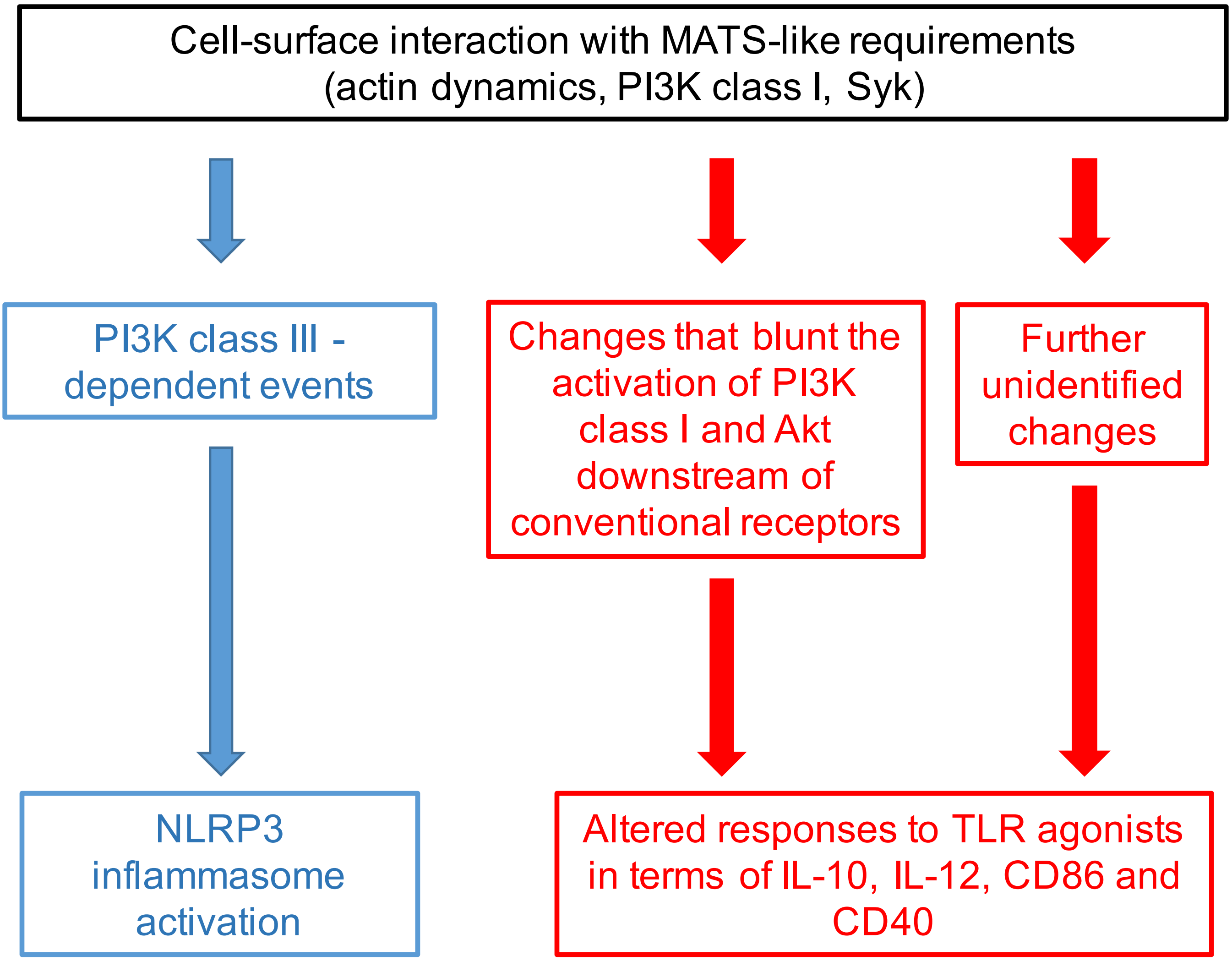

Suppl. Fig. 7. Summary of proposed mechanisms in BMDC responses to pLL. The diagram is based on results from the present and a previous paper (Ref 36). The cell-surface interaction necessary for all responses observed probably arises in a context of frustrated phagocytosis, and has MATS-like requirements. In the context of this interaction different signaling mechanisms are triggered. One of these, which may require the development of digestive exophagy and therefore needs PI3K class III, leads to NLRP3 inflammasome activation (blue). The other signaling mechanisms, which do not require PI3K class III, lead to NLRP3-independent alterations in IL-10, IL-12, CD86 and CD40 responses to LPS (red); a branch of these causes the blunted activation of Akt in response to ligation of receptors that couple to PI3K class I. Although the overall interaction and the whole set of responses analyzed have MATS-like requirements, we consider the possibility that MATS signalling in strict terms (i.e. involving the recruitment of Syk to moesin, as described in Ref 41) is responsible for only a subset of these responses. In this context, Syk may be involved in establishing the synapse with the particles and additionally in some, but not necessarily all, of the signaling mechanisms that follow.
